# Frequency and clinical relevance of potential cytochrome P450 drug interactions in a psychiatric patient population – an analysis based on German insurance claims data

**DOI:** 10.1186/s12913-016-1724-8

**Published:** 2016-09-08

**Authors:** Julia K. Ostermann, Anne Berghöfer, Frank Andersohn, Felix Fischer

**Affiliations:** 1 Institute for Social Medicine, Epidemiology, and Health Economics, Charité - Universitätsmedizin Berlin, Luisenstrasse 57, 10117 Berlin, Germany; 2 Department of Psychosomatic Medicine, Center for Internal Medicine and Dermatology, Charité - Universitätsmedizin, Berlin, Germany

**Keywords:** Secondary data analysis, Health claims data, CYP450, Drug-drug exposure, Drug-drug interaction, Antidepressants, Antipsychotics

## Abstract

**Background:**

Numerous drugs used in the treatment of psychiatric disorders are substrates of cytochrome P450 enzymes and are potential candidates for drug-drug interactions (DDIs).

**Methods:**

Claims data of a German statutory health insurance company from severely mentally ill patients who registered in an integrated care contract from August 2004 to December 2009 were analysed. We measured time periods of concomitant prescription of drugs that have been reported to interact via cytochrome P450, with a focus on drugs acting as strong inhibitors. Such drug-drug exposure (DDE) is an incontrovertible precursor of DDIs. We assessed whether potential DDIs were considered clinically relevant based on the prescribing information of the respective drugs.

**Results:**

Among all 1221 patients, 186 patients (15.2 %; Clopper-Pearson 95 % confidence interval (CI): 13.3–17.4 %) had at least one DDE prescription, and 58 patients (4.8 %; 95 % CI 3.6–6.1) had at least one DDE prescription involving a strong cytochrome P450 inhibitor. In 59 patients, (4.8 %; 95 % CI: 3.7–6.2 %) five or more DDEs were identified, and five or more DDEs with a strong inhibitor were identified in 18 patients (1.5 %; 95 % CI: 0.9–2.3). The rates of DDEs were 0.27 (Garwood 95%CI: 0.25–0.28) per person-year and 0.07 (95 % CI: 0.07–0.08) for strong-inhibitor DDEs. Four of the ten most frequent DDEs were identified as clinically relevant, and seven of the eight most frequent DDEs involving a strong inhibitor were clinically relevant.

**Conclusions:**

The number of patients with DDEs was not alarmingly high in our sample. Nevertheless, prescription information showed that some prescribed drug combinations could result in serious adverse consequences that are known to weaken or strengthen the effect of the drugs and should therefore be avoided.

## Background

Pharmacokinetic drug-drug interactions (DDIs) indicate the influence that one drug has on the blood concentration of another drug [1–3]. The risk of DDIs is increased in psychiatric patients [4], as they are often prescribed several concurrent long-term medications [5–8]. In many cases, several physicians treat these patients, which presents a challenge in the medical care of this patient population because the German health system is divided into outpatient (primary and secondary care) and inpatient care. Therefore, mental health specialists may not perceive somatic co-morbidities in psychiatric patients. Thus, monitoring of DDIs in this specific population is particularly relevant.

In this study we focussed on potential DDIs involving cytochrome P450 (CYP450) enzymes, as CYP450 enzymes metabolise many drugs. Hence, DDIs are likely to occur [9–12]. Six CYP450 enzymes are involved in the metabolism of approximately half of all drugs, which emphasises the importance of CYP450s in the analysis of DDIs [13]. CYP450 genes [2, 12–15] are subdivided into different families and subtypes according to their shared amino acid identity [16]. Drugs can act as substrates, inhibitors, or inducers of CYP450 enzymes [2]. Drugs that act as inhibitors may reduce or disable enzymatic activity, whereas inducers increase enzymatic activity [17]. Therefore, we examined the reciprocal CYP450 interactions of inhibitors and substrates and inducers and substrates. Inhibitors are classified as ‘low’, ‘moderate’, ‘strong’ or ‘without specification’ based on whether their plasma AUC values increase or clearance decreases [18]. However, the majority of potential DDIs experienced by patients may not result in clinically relevant interactions [3]. A prerequisite for the occurrence of DDIs is the time period of concurrent exposure to potentially interacting prescribed drugs (drug-drug exposures (DDEs)). DDEs influence the patient’s risk of developing DDIs [19]. It can be assumed that patients with a psychiatric illness are particularly prone to DDEs and, hence, DDIs because they are often prescribed multi-drug regimens [6]. A British survey conducted at a psychiatric ward indicated that 19 % of the patients were prescribed a potentially clinically important CYP2D6 combination, and 6 % were prescribed a potentially clinically important CYP3A4 combination [6]. According to one review, although millions of patients taking antidepressants experience DDEs, the prevalence of clinically significant DDIs is not clear [20]. In this context, health insurance data can be utilised to investigate the prevalence of DDEs because all medications prescribed in the outpatient sector are documented for reimbursement purposes. Thus, we investigated whether drug-prescribing behaviour poses a relevant hazard for DDIs in psychiatric outpatient care by using health claims data from mentally ill patients from an integrated care contract. As DDEs are an incontrovertible precursor of DDIs, we calculated the frequency of concurrent drug prescriptions and assessed the clinical relevance of the potential DDIs in association with prescription information for the drugs.

## Methods

### Data source

Clinical and sociodemographic data were prospectively collected in an observational study evaluating an integrative outpatient treatment model for seriously mentally ill patients in Berlin, Brandenburg, and Lower Saxony. Patients entered the study on July 1, 2004, or at the time of their entry into the integrative outpatient treatment model, whichever occurred later. All patients were followed up until the end of their membership in the integrative outpatient treatment model, death, or the end of the study period (December 31, 2009), whichever came first (see Fischer et al. for details regarding the study design and participants [21]). The study was approved by the Ethics Committee of the Charité-Universitätsmedizin Berlin (EA1/088/08). All patients analysed were insured under the same statutory health insurance (Deutsche Angestelltenkrankenkasse, ‘DAK’) so that claims data could be used for this study. Permission to use the claims data was obtained from the statutory health insurance. Available data included demographics, clinical information from psychiatric diagnoses, and information on all reimbursed outpatient prescriptions. Prescription data included the anatomical-therapeutic chemical (ATC) code, which is used to classify substances, and the prescription date for every prescribed drug.

### Definition of a potential interaction

**Fig. 1:**
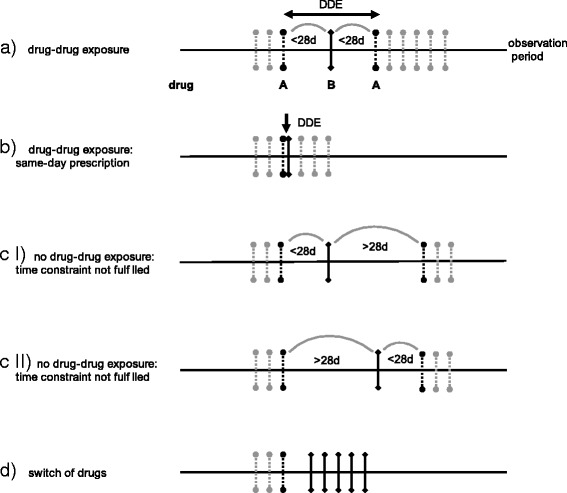
Title: Definition of a DDE. Legend: *Dotted line* represents the victim drug A. *Solid line* represents perpetrator drug B. *Black arrows* point to relevant drugs for DDE definitions. **a** Definition of a DDE. Drug B is prescribed between prescriptions of drug A within a 28-day time interval. **b** Exceptional case of DDE definition: drugs A and B are prescribed on the same day. **c** I no DDE by definition: drug A is prescribed, within a 28-day interval drug B is prescribed, but the next prescription of drug A exceeds the 28-day interval. **c** II no DDE by definition: drug A is prescribed, the prescription of drug B exceeds the 28-day interval, and the next prescription of drug A lies within the 28-day interval. **d** no DDE: drug A is prescribed, then the patient is switched to drug B. No prescription of drug A re-occurs

To define possible inhibitors, inducers and substrates of CYP450 enzymes from the prescription data, David Flockhart’s clinically relevant drug interaction table for CYP450 interactions was used, as it is one of the most commonly employed data sources for identifying drug interactions via CYP450s and their clinical relevance (http://medicine.iupui.edu/clinpharm/ddis/clinical-table/) [18]. All CYP450-relevant drugs in Flockhart’s table are classified into inhibitors, inducers or substrates of specific isoenzymes (1A2, 2B6, 2C8, 2C9, 2C19, 2D6, 2E1 or 3A4, 5, 7). Some drugs act as substrates or inhibitors of CYP450 enzymes, depending on the interactions of the victim drug. These drugs were classified as both a substrate and an inhibitor. We searched all prescriptions successively in chronological order to identify DDEs in the data. Each entry successively served as the perpetrator drug. We extracted the prescription date, the CYP450 isoform, and whether the drug acts as a substrate, inducer or inhibitor of CYP450 enzymes from each perpetrator drug. A DDE was defined as the prescription of an perpetrator drug (e.g., inhibitor) of a specific CYP450 isoform (e.g., 2D6) between prescriptions of a DDE victim drug (e.g., substrate) of the same CYP450 isoform if the time-span between the prescription of the two drugs did not exceed 4 weeks (Fig. 1 a). The 4-week timeframe is consistent with other claims data studies [22, 23] and we are certain that the probability of both drugs taken concomitantly is relatively high. Therefore, an perpetrator drug must be bracketed by a victim drug (‘victim drug’–‘perpetrator drug’–‘victim drug’). One exception to this definition is same-day prescriptions in which the perpetrator drug was not bracketed by the victim drug (Fig. 1 b). The prescription scheme was not defined as a DDE if the time-span between the prescriptions exceeded 4 weeks (Fig. 1 c I-II). Our DDE definition that an perpetrator drug must be prescribed between prescriptions of a victim drug was important to exclude changes in drugs (switches), which were defined as changes to a medication plan for a patient (Fig. 1 d).

### Statistical analysis

Demographic and medical data of the study participants were analysed descriptively. For proportions, Clopper-Pearson 95 % confidence intervals (CIs) were calculated, and for rates, Garwood 95 % CIs were calculated because for high and low proportions in the data, these assumptions are a better approximation than the Gaussian assumption [24]. The most commonly prescribed drugs and the most commonly prescribed drugs that act on CYP450 enzymes were identified using the therapeutic/pharmacological subgroups (level 3) of the ATC system. The number of patients who were prescribed a drug scheme that met our DDE definition was counted to estimate the number of patients who potentially experienced at least one DDE within the observation period. The number of patients who potentially experienced at least one DDE with a strong inhibitor was counted to account for potentially more clinically relevant DDEs.

The observation period varied among patients depending on the individual duration of the integrative outpatient treatment model. Therefore, DDEs per person-year were calculated. The time at risk was calculated as the time-span between the first and last occurrence dates in the data for each patient. Dates of drug prescriptions, physician contacts, hospital admission and rehabilitation were included in calculations of the total time interval for each patient. Patients are not at risk for drug prescriptions during a hospital or rehabilitation stay. Patients get their drugs directly from the hospital or rehabilitation centre and due to the lump-sum based reimbursement system in the German inpatient care sector, these drugs are not included in the health claims data. Therefore, time intervals for hospital or rehabilitation stays were subtracted from the total time at risk. The mean duration until the occurrence of a prescription with a DDE was calculated in all patients with at least one DDE prescription. The mean duration until the event was defined as the average time-span between the first occurrence in the data and the first DDE prescription scheme.

DDE combinations that were prescribed most often in our sample were identified in a first step for all potential interactions and in a second step for all potential interactions with strong inhibitors. DDE combinations were analysed with respect to the number of patients who were prescribed this combination, the DDEs per person-years and their clinical relevance. The prescribing information for brand-name versions of the perpetrator drug and the interaction victim were screened to evaluate whether it was advised that the drugs should not be taken together and whether this DDE was mentioned as potentially leading to a DDI (i.e., “clinically relevant”). The impacts of sex and age were evaluated by calculating stratified DDE exposure rates.

All statistical analyses were performed using R version 3.0.1 [25]. Confidence intervals for rates and proportions were calculated with the ‘exactci’ package [24].

## Results

### Participants

Data from 1221 patients (846 females) with a mean age of 47.9 years (standard deviation (SD) 16.2) were available for analysis. Most patients were diagnosed with depression (*n* = 338, 28 %), recurrent depression (*n* = 304, 24 %), or schizophrenia (*n* = 163, 13 %). Patients had a mean observation period of 4.3 years (SD 1.2) (Table 1).

**Table 1:** Baseline characteristics of all patients (*n* = 1221)

	All patients (*n* = 1221)
Female, n (%)	846 (69.3)
Age, mean (SD)	47.9 (16.2)
Younger than 35 years, n (%)	269 (22.0)
35–55 years old, n (%)	592 (48.5)
Older than 55 years, n (%)	360 (29.5)
Most common psychiatric diagnosis, n (%)	
F32 – Major depressive disorder	338 (27.7)
F33 – Major depressive disorder, recurrent	304 (24.9)
F20 – Schizophrenia	163 (13.4)
F41 – Anxiety disorders	96 (7.9)
F31 – Bipolar disorder	66 (5.4)

### Prescriptions

Patients were prescribed 17.1 drugs on average (95 % confidence interval (CI): 17.0–17.3) per person-year. Most patients were prescribed antidepressants, antibacterials and antipsychotics (Table 2). The most commonly prescribed antidepressants were citalopram (*n* = 436, 35.7 %) and mirtazapine (*n* = 314, 25.7 %). The most commonly prescribed antibacterials were amoxicillin (*n* = 267, 21.9 %) and doxycycline (*n* = 236, 19.3 %). Lorazepam (*n* = 287, 23.5 %) and quetiapine (*n* = 203, 16.6 %) were the most commonly prescribed antipsychotics. The most commonly prescribed drugs that act on CYP450 enzymes included anti-inflammatory/antirheumatic products, antidepressants and antipsychotics (Table 3). Ibuprofen (*n* = 503, 41.2 %) and diclofenac (*n* = 450, 36.9 %) were the most commonly prescribed anti-inflammatory/antirheumatic drugs. The most frequently prescribed antidepressants were venlafaxine (*n* = 227, 18.6 %) and doxepin (*n* = 183, 15.0 %). Risperidone (*n* = 164, 13.4 %) and olanzapine (*n* = 154, 12.6 %) were the most commonly prescribed antipsychotics.

**Table 2:** Most frequently prescribed drugs by ATC codes

	All patients (*n* = 1221)	
Most commonly prescribed drugs by ATC codes	Single prescriptions	Number of patients (%)
N06 – Antidepressants	14,001	973 (79.7)
J01 – Antibacterials	3812	885 (72.5)
N05 – Antipsychotics	20,021	844 (69.1)
M01 – Anti-inflammatory/antirheumatic	3728	740 (60.6)
N02 – Analgesics	5996	553 (45.3)
A02 – Drugs for acid-related disorders	3333	475 (38.9)
A03 – Drugs for functional gastrointestinal disorders	1222	366 (30.0)
D07 – Corticosteroids	902	324 (26.5)
R06 – Antihistamines	1573	317 (26.0)
C07 – Beta-blocking agents	2867	307 (25.1)
…		
Sum	89,361	N/A

The relative frequency (n) and number of patients who were prescribed the drug (n, %) are shown. Note that patients could be prescribed several drug classes

**Table 3:** Most frequently prescribed drugs that interact with CYP450 enzymes by ATC codes

	All patients (*n* = 1221)	
Prescribed drugs by ATC codes	Frequency	Number of patients (%)
M01 – Anti-inflammatory/antirheumatic products	3313	713 (58.40)
N06 – Antidepressants	6249	635 (52.0)
N05 – Antipsychotics	5879	471 (38.58)
A02 – Drugs for acid-related disorders	3050	448 (36.69)
N02 – Analgesics	1473	250 (20.48)
J01 – Antibacterials for systemic use	247	146 (11.96)
C10 – Lipid-modifying agents	941	137 (11.22)
C08 – Calcium channel blockers	1195	131 (10.73)
R05 – Cough and cold preparations	165	100 (8.19)
N03 – Antiepileptics	523	80 (6.55)
…		
Sum	25,211	N/A

The relative frequency (n) and number of patients who were prescribed the drug (n, %) are shown. Note that patients could be prescribed several drug classes

### Number of DDEs

A total of 1122 patients (91.9 %) in our sample were prescribed drugs that may act on CYP450 enzymes. During the entire 5-year observation period, 186 patients (15.2 %; 95 %–CI: 13.3–17.4) exhibited at least one DDE prescription scheme as described above. At least three DDE prescription schemes were identified in 91 patients (7.5 %; 95 %–CI: 6.0–9.1), and at least five DDE prescription schemes were identified in 59 patients (4.8 %; 95 %–CI: 3.6–6.1). Prescription of at least one DDE with a strong inhibitor was observed in 58 patients (4.8 %; 95 %–CI: 3.6–6.1). At least three DDE prescription schemes with a strong inhibitor were identified in 27 patients (2.2 %; 95 %–CI: 1.5–3.2), and at least five DDE prescription schemes with a strong inhibitor were discovered in 18 patients (1.5 %; 95 %–CI: 0.9–2.3).

### Person-time analyses

A total of 1393 DDEs were identified in all 1221 patients. The total observation time for all patients was 5221.3 years. We subtracted 8.4 years of hospital time during which patients were not at risk of being prescribed potentially interacting drugs in the outpatient sector. Consequently, the total person-time at risk was 5212.8 years. Therefore, the number of prescriptions of DDEs was 0.27 (95 %–CI: 0.25–0.28) per person-year. Males had a higher number of prescriptions of DDEs per person-year than females (males: 0.32, 95 %–CI: 0.30–0.35; females: 0.24, 95 %–CI: 0.23–0.26). Patients aged 40–65 years had the highest number of DDE prescriptions among the three age categories (0.34, 95 %–CI: 0.32–0.36). Patients younger than 40 years or older than 65 years had similar numbers of DDE prescriptions (younger: 0.18, 95 %–CI: 0.16–0.20; older: 0.18, 95 %–CI: 0.16–0.22). Males aged 40–65 years had a higher number of prescriptions of DDEs per person-year (0.46, 95 %–CI: 0.41–0.51) than younger (<40 years; 0.14, 95 %–CI: 0.12–0.18) or older (>65 years; 0.29, 95 %–CI: 0.22–0.37) males. Females aged 40–65 years had a higher number of prescriptions of DDEs per person-year (0.29, 95 %–CI: 0.27–0.31) than younger (<40 years; 0.20, 95 %–CI: 0.17–0.23) or older (>65 years; 0.15, 95 %–CI: 0.13–0.19) females.

The total years to event (prescription of potentially interacting drugs) were 402.1 years in all 186 patients who experienced a DDE. Therefore, the duration until the event was 2.16 years on average (SD 1.41).

### Most commonly recorded potential interactions

The most common pairs of prescribed drugs identified as DDEs were diazepam and omeprazole, doxepin and venlafaxine, and doxepin and paroxetine (Table 4). Four of the ten most frequent DDEs were evaluated as clinically relevant according to the prescribing information. The most commonly prescribed drug combinations with a strong inhibitor were amitriptyline and paroxetine and paroxetine and risperidone (Table 5). The eight most frequent DDEs were identified, and seven of these eight were evaluated as clinically relevant. According to the prescribing information, concomitant intake of these drugs should be avoided.

**Table 4:** Most frequent potential drug-drug exposures. The classification of a drug-drug exposure was determined by the prescription information for the brand-name drugs

Potential drug-drug exposure	Frequency	Events per 100 person-years (Poisson exact 95 %–CI)	Number of patients with at least one DDE (%)	Clinical relevance of a potential interaction (as per prescribing information)
diazepam & omeprazole	52	1.00 (0.74–1.31)	17 (1.39)	Omeprazole may increase systemic exposure to diazepam [31]
doxepin & venlafaxine	54	1.03 (0.78–1.35)	15 (1.23)	not mentioned
doxepin & paroxetine	44	0.84 (0.61–1.13)	13 (1.06)	not mentioned
amitriptyline & omeprazole	58	1.11 (0.84–1.44)	12 (0.98)	not mentioned
doxepin & tramadol	51	0.98 (0.73–1.28)	11 (0.90)	Tramadol may increase the potential of seizures related to tricyclic antidepressants. Serotonin syndrome may occur [32] [unclear if due to CYP interactions]
amitriptyline & paroxetine	29	0.56 (0.37–0.80)	9 (0.74)	Patients taking SSRIs should only be treated with amitriptyline with particular caution [33] [reason not given]
amitriptyline & esomeprazole	31	0.59 (0.40–0.84)	8 (0.66)	not mentioned
doxepin & risperidone	95	1.82 (1.47–2.22)	8 (0.66)	Mutual reinforcement of the central depressant effect [34]
fluoxetine & omeprazole	11	0.21 (0.11–0.38)	8 (0.66)	not mentioned
doxepin & duloxetine	22	0.42 (0.26–0.64)	7 (0.57)	not mentioned
…				
Sum	1393	26.72 (25.34–28.16)	330

**Table 5:** Most frequent drug-drug exposures with a strong inhibitor in all patients (*n* = 1221). Classification of the potential drug-drug exposure was determined by the content of the prescribing information associated with the brand-name drug

Potential drug-drug exposure (bold = strong inhibitor)	Frequency	Events per 100 person-years (95%CI)	Number of patients with at least one DDE (%)	Clinical relevance of a potential interaction (as per prescribing information)
amitriptyline & paroxetine	29	0.56 (0.37–0.80)	9 (0.74)	Patients taking SSRIs should only be treated with amitriptyline with particular caution [35] [reason not given]
paroxetine & risperidone	21	0.40 (0.25–0.62)	6 (0.49)	Paroxetine increases the plasma-concentration of risperidone [36]
codeine & fluoxetine	8	0.15 (0.07–0.30)	5 (0.41)	not mentioned [37, 38]
amitriptyline & fluoxetine	16	0.31 (0.18–0.50)	5 (0.41)	Taking fluoxetine and amitriptyline in parallel might result in an increased plasma-concentration of amitriptyline Dose-reduction might be necessary [33]
fluoxetine & tramadol	42	0.81 (0.58–1.09)	5 (0.41)	Taking tramadol and fluoxetine in parallel can induce serotonin syndrome [32]
amlodipine & clarithromycin	5	0.10 (0.03–0.22)	4 (0.33)	Taking clarithromycin and amlodipine parallel might result in an increased plasma concentration of amlodipine [39]
clomipramine & paroxetine	76	1.46 (1.15–1.82)	4 (0.33)	Paroxetine can increase the plasma concentration of clomipramine [40]
paroxetine & tramadol	6	0.12 (0.04–0.25)	4 (0.33)	Taking tramadol and SSRIs [i.e., paroxetine] in parallel can induce serotonin syndrome [32]. Patients taking tramadol and paroxetine must be monitored closely [35]
…				
Sum	380	7.29 (6.57–8.06)	90	

## Discussion

### Key results

Our analyses revealed that 15 % of patients were prescribed a combination of drugs imposing a risk of DDI at least once; in approximately 5 % of patients, this combination included a strong inhibitor. Several of the drug combinations that we identified may cause relevant interactions. The data suggests that in most of our patient cases the intended effect of the drugs will be strengthening.

### Interpretations

Other studies have indicated that the proportion of psychiatric patients at risk of potential DDIs via DDE ranges from approximately 23 % (Guo et al.) [26] to 28 % (Davies et al.) [6]. In our sample, 15.3 % of the patients were prescribed at least one drug combination classified as a DDE. The differences in these numbers may be attributed to the different methodologies used in these studies. Davies et al. performed a cross-sectional study in an inpatient psychiatric ward, while Guo et al. used Medicaid claims data over 4 years and primarily focused on the antipsychotics haloperidol, perphenazine, and chlorpromazine. However, these authors did not include a longitudinal analysis. The proportion of DDEs per person-year in our analysis was approximately one-third. Therefore, our assessment of potential DDEs in mentally ill patients suggests that the prevalence of DDIs in our sample is not alarmingly high. However, several of the identified DDEs are known to lead to clinically relevant DDIs that should be avoided. Psychiatric patients are often treated across care sectors, which may result in a lack of communication and information transfer. This lack of communication may lead to changes in medication plans and inappropriate drug prescriptions that increase the patients’ risk of DDIs [27]. One Swiss study noted that patients are often discharged from hospitals with prescribed drug combinations that may lead to DDEs or DDIs [28]. One survey indicated that adverse effects due to DDIs are frequently encountered in the outpatient sector [29]. German GPs do not act as gatekeepers, and patients can contact their preferred specialists themselves. A GP prescribing clarithromycin to a patient might not know that the patient’s long-term medication plan includes diazepam prescribed by a mental health specialist. This lack of communication may increase the patient’s risk for relevant DDIs. Therefore, our analyses of psychiatric patients are relevant for the estimation of DDEs, despite the fact that we only covered outpatient care. Notably, we identified several combinations of DDEs prescribed by the same mental health specialist, which is in accord with the findings of Guo et al. [26]. Initially, we would have assumed that most DDEs arise from drugs of different classes that are prescribed by several physicians who are unknown to the other prescribing physicians.

### Strengths and limitations

One particular strength of our secondary data analyses is that we were able to analyse data from many patients using their complete prescription records for a long observation period. The comprehensiveness of these data provides a complete picture of drug prescriptions for each insured person, which results in a precise picture of DDEs. The numbers of identified DDEs were not alarmingly high in our sample, but our analyses only covered estimations of pharmacokinetic DDEs caused by drugs that are metabolised by CYP450 enzymes. Therefore, the number of DDEs and potential DDIs that are not due to CYP450 enzymes in our sample is likely to be even higher and more relevant than we estimated.

A major limitation of our analyses is that we do not have available data to establish the real linkage between DDEs and DDIs in the patients. This is because the health claims data only included information about the medication prescribed and the prescription date. Hence, the actual intake of the drugs, effects on biological availability and possible medical outcomes could not be assessed. Therefore, it is unclear whether the DDEs identified based on our definition led to actual simultaneous intake of the drugs and eventually to DDIs. However, even with available medical outcome data, it would be difficult to identify DDIs beyond severe reactions that require medical treatment, as patients experiencing a DDI might not consult a physician but instead stop taking one of the drugs. A further limitation is that the estimation of DDEs might be imprecise because data on drug use during inpatient stays and on drugs bought over the counter are missing. Moreover, specific information on the dosage form or packaging size of the prescribed drugs was not available. Dosage information might be important for estimating the clinical relevance of DDIs more precisely. In future studies, use of the ‘Pharmazentralnummer’ (PZN; central pharmaceutical number) could allow more accurate calculations of exposure dates to be made based on the packaging size, as compared with ATC codes. A US study postulated that patients must be exposed to two drugs in parallel for at least ten days to identify potential DDIs in pharmacy claims data [30]. Unfortunately, we were unable to verify this constraint because the PZN was not included in the claims data that we received for the study patients. However, the use of a time constraint of only 4 weeks and the fact that many psychiatric patients are constantly on medications increases our confidence that our analyses provide indications of potential DDIs. The specific disease profiles in our sample limit the generalisability of our results to the general population, and similar analyses in other high-risk groups (e.g., patients with chronic conditions or the elderly) are needed.

### Outlook

DDEs are potentially hazardous to patient health in fragmented health care systems because of the lack of information sharing among care sectors. Secondary data analyses seem promising for the identification of DDEs and potential DDIs across care sectors, which could reduce health care utilisation and costs as a result of (avoidable) drug interactions. However, DDIs are difficult to identify in claims data unless an adverse event occurred and was documented. Claims data must be merged with patient outcome data to validate whether our definition of DDEs can be used to identify other DDIs beyond events requiring hospitalisations. Case managers of health insurance organisations could use the DDE definition to screen the prescription data of their insured persons and determine who is at risk of experiencing DDIs. These insured persons (or their physicians) could then be advised of possible drug alternatives to avoid DDEs. Costs of outpatient care or hospital admissions due to DDIs could potentially be reduced, which would be of (financial) interest to health insurance companies. Claims data that include relevant prescription information could be stored on health insurance cards. The health insurance card could also store long-term use of over-the-counter drugs if the GP or pharmacy records these drugs. This information should be linked with existing interaction databases on a regular basis to recognise potential DDIs. A comprehensive picture of drug use could prevent clinically relevant DDIs if data protection can be fully ensured.

## Conclusion

Our data suggest that the numbers of patients with clinically relevant DDIs in our sample is not alarmingly high. Nevertheless, some prescribed drug combinations are to be avoided as they might lead to serious adverse consequences. An improved utilisation of claims data would allow for an examination of DDEs in routine clinical practice and provide an opportunity to potentially implement warning systems for DDIs in real-life settings to optimally minimise the number of patients at risk.

## Abbreviations

ATC code: Anatomical-therapeutic chemical code
CYP450: Cytochrome P450
DDE: Drug-drug exposure
DDI: Drug-drug interaction
PZN: Pharmazentralnummer (central pharmaceutical number)
